# Total thyroidectomy without prophylactic central neck dissection in clinically node-negative papillary thyroid cancer: is it an adequate treatment?

**DOI:** 10.1186/1477-7819-12-152

**Published:** 2014-05-20

**Authors:** Pietro Giorgio Calò, Giuseppe Pisano, Fabio Medas, Jacopo Marcialis, Luca Gordini, Enrico Erdas, Angelo Nicolosi

**Affiliations:** 1 Department of Surgical Sciences, University of Cagliari, Cagliari, Italy

**Keywords:** Papillary thyroid carcinoma, Central neck dissection, Total thyroidectomy

## Abstract

**Background:**

Cervical lymph node metastases in papillary thyroid cancer are common. Although central neck dissection is indicated in clinically nodal-positive disease, it remains controversial in patients with no clinical evidence of nodal metastasis. The aim of this retrospective study was to determine the outcomes of clinically lymph node-negative patients with papillary thyroid cancer who underwent total thyroidectomy without a central neck dissection, in order to determine the rates of recurrence and reoperation in these patients compared with a group of patients submitted to total thyroidectomy with central neck dissection.

**Methods:**

Two-hundred and eighty-five patients undergoing total thyroidectomy with preoperative diagnosis of papillary thyroid cancer, in the absence of suspicious nodes, were divided in two groups: those who underwent a thyroidectomy only (group A; n = 220) and those who also received a central neck dissection (group B; n = 65).

**Results:**

Six cases (2.1%) of nodal recurrence were observed: 4 in group A and 2 in group B. Tumor histology was associated with risk of recurrence: Hürthle cell-variant and tall cell-variant carcinomas were associated with a high risk of recurrence. Multifocality and extrathyroidal invasion also presented a higher risk, while smaller tumors were at lower risk.

**Conclusions:**

The role of prophylactic central lymph node dissection in the management of papillary thyroid cancer remains controversial. Total thyroidectomy appears to be an adequate treatment for clinically node-negative papillary thyroid cancer. Prophylactic central neck dissection could be considered for the more appropriate selection of patients for radioiodine treatment and should be reserved for high-risk patients only. No clinical or pathological factors are able to predict with any certainty the presence of nodal metastasis. In our experience, tumor size, some histological types, multifocality, and locoregional infiltration are related to an increased risk of recurrence. The potential use of molecular markers will hopefully offer a further strategy to stratify the risk of recurrence in patients with papillary thyroid cancer and allow a more tailored approach to offer prophylactic central neck dissection to patients with the greatest benefit. Multi-institutional larger studies with longer follow-up periods are necessary to draw definitive conclusions.

## Background

Papillary thyroid carcinoma (PTC) is the most common thyroid malignancy, comprising approximately 90% of new cases of thyroid cancer in iodine-sufficient areas of the world [1-6]. Patients with PTC have an excellent prognosis, with 10-year survival rates exceeding 90% [1,2,4,6,7]. Total thyroidectomy (TT) is generally accepted as the procedure of choice for all PTC exceeding 10 mm in diameter [1,2,8].

Despite the excellent prognosis, cervical lymph node metastases are common, occurring in 20 to 50% of patients [1,2,6,8-14]. Micrometastases are even more common and may be found in 90% of patients [9-11,14,15]. The most common sites of metastases are the central neck lymph nodes (level VI) [1,2,6,9,12,16-18]. Nodal metastases are associated with an increase in recurrence rate and may impact negatively on survival [2,9,11,19,20].

Although central neck dissection is indicated in clinically nodal-positive disease, it remains controversial in patients with no clinical evidence of nodal metastasis [5,21].

Some authors recommend routine central neck dissection in order to prevent a future recurrence, citing the high risk of positive lymph nodes, the accuracy of staging, better outcomes, reduced postoperative thyroglobulin (Tg) levels, and a lower morbidity rate associated with the first operation [14,15,18,19,22-26], whereas others suggest that this procedure increases the risk of injury to parathyroid glands and recurrent laryngeal nerves, without any demonstrable benefits in terms of long-term survival [2,10,11,15,18,22,23,27],[28].

Because of the risk of complications following prophylactic central neck dissection, recently several authors have introduced a more limited (ipsilateral) central neck dissection, as an alternative treatment for patients with unilateral PTC [20,25,29].

The aim of this retrospective study was to determine the outcomes of clinically lymph node- negative patients with PTC who underwent total thyroidectomy without a central neck dissection, in order to determine the rates of recurrence and reoperation in these patients compared with a group of patients submitted to total thyroidectomy with central neck dissection.

## Methods

The clinical records of patients undergoing total thyroidectomy with or without prophylactic lymphadenectomy, between 2002 and 2010, presenting preoperative cytological evidence of PTC but no signs of enlarged lymph nodes during preoperative ultrasonography and intraoperative inspection and palpation were analyzed. In our unicenter retrospective study, 285 consecutive PTC patients were identified by computerized search. These patients had undergone TT with curative intent in our Department of Surgical Sciences at the University of Cagliari. The data were collected from retrospective chart review. Patients were divided into two groups: those who had received a thyroidectomy without central neck dissection (group A; n = 220), and those who had received a thyroidectomy with central neck dissection (group B; n = 65). These patients were considered for central neck dissection on the basis of the surgeon’s judgment of the surgical field, macroscopic appearance of the tumor, tumor size, and suspicion of extra-capsular invasion. In all cases, surgery was performed by three experienced endocrine surgeons. For each patient, a preoperative diagnosis of PTC had been obtained by ultrasound (US)-guided fine-needle aspiration cytology (FNAC). The preoperative work-up consisted of free thyroid hormone (FT3, FT4), thyrotropin (TSH), Tg and anti-Tg antibody (TgAb) measurements, and high resolution US of the neck by a skilled sonographer. A pre- and postoperative fibrolaryngoscopy was routinely performed in all patients. Postoperative diagnosis of lymph node recurrence was performed by US-guided FNAC and Tg measurement in FNAC fluid wash-out (FNAC-Tg) in the case of enlarged lymph nodes ≥ 1 cm. Patient demographics and postoperative complications were recorded, including neck hematomas requiring reoperation, transient or permanent hypoparathyroidism, transient or permanent vocal cord palsy, and distant and locoregional recurrence detected by postoperative surveillance. Hypoparathyroidism (defined as a parathyroid hormone (PTH) level < 10 pg/ml; normal values range between 10 to 65 pg/ml) was considered permanent when it lasted for more than six months. Permanent recurrent laryngeal nerve injury was defined as vocal fold paralysis, confirmed with laryngoscopic examination, persisting for more than six months. Qualitative data were expressed as percentages, while quantitative data were expressed as the mean value ± standard deviation.

The study was approved by the Institutional Ethical committee of the University of Cagliari.

### Biochemical assays

FT3, FT4, and TSH were determined by automatic ultrasensitive chemiluminescent assays (Ortho Clinical Diagnostic SpA, Milan, Italy). Tg and TgAb were detected by chemiluminescent assays (Immulite 2000, Diagnostic Products Corporation, Los Angeles, CA, USA; distributor Medical Systems Corporation, Genoa, Italy).

### Surgery

All operations were performed by the same three surgeons, thus the TT and lymph node dissection techniques used were consistent across patients. Recurrent laryngeal nerves were routinely identified and exposed up until their insertion in the larynx, and parathyroid glands were identified and preserved. In cases of suspected devascularized or incidentally removed parathyroid glands, a muscular autoimplantation followed. Serum calcium and intact parathyroid hormone levels were assayed on the first postoperative day, and subsequently on the basis of a clinical evaluation.

### Follow-up

Patient follow-up examinations consisted of neck USs and the monitoring of serum Tg and TgAb levels every six months during suppressive L-thyroxine treatment. A serum Tg level ≤ 0.2 ng/ml was taken as undetectable. Surveillance for possible recurrence in patients considered disease-free was achieved by Tg detection or, in those positive for TgAb, by whole-body ^131^I scanning after recombinant human thyrotropin (rhTSH) stimulation and neck US. Diagnosis of disease recurrence in the cervical lymph nodes was based on US-guided FNC, Tg washing of FNC aspirates, and serum Tg level monitoring. The median length of follow-up was 100 months (range 38 to 137 months).

### Statistical analysis

Data were analyzed using descriptive statistics: for categorical variables, the Pearson’s chi-squared (exact) test was used; for quantitative variables independent Student’s *t*-tests were used. Data were reported as the mean value ± standard error of the mean (SEM). All calculations were performed using the software package SPSS 15.0 for Windows (SPSS Inc., Chicago, IL, USA). Comparisons were considered statistically significant for *P* < 0.05.

## Results

Between September 2002 and December 2010, 285 PTC patients, 225 women and 60 men (F/M ratio = 3.75/1), with a median age of 51.5 years (range 18 to 81), were submitted to TT. Group A consists of 220 patients, 172 female and 48 male (F/M ratio = 3.58/1) with a median age of 53 years (range 18 to 81), who were submitted to TT without lymphadenectomy. Group B consists of 65 patients, 53 female and 12 male (F/M ratio = 4.41/1) with a median age of 46 years (range 20 to 77), who were submitted to TT with prophylactic central neck bilateral lymphadenectomy (see Table 1). Median nodal yield for patients undergoing central neck dissection was 7.5. A neck hematoma, requiring surgical re-exploration was observed in 3 patients (1.05%): 2 in group A (0.9%) and one in group B (1.53%) (*P* = 0.79). Transient or definitive hypoparathyroidism was observed in 55 (25%) and 10 patients (4.54%), respectively, in group A; while 25 (38.46%) and 7 patients (10.76%) were diagnosed in group B, respectively (*P* = 0.049 and 0.117). Temporary recurrent laryngeal nerve paralysis was observed in 3 patients in group A (1.36%) and in 2 patients in group B (3.07%) (*P* = 0.69). No cases of permanent or bilateral recurrent laryngeal paralysis were observed (see Table 2).

**Table 1:** Demographic and pathological data of 285 papillary thyroid cancer patients

	**Total**	**Group A**	**Group B**	***P***
Patients	285	220	65	
Male	60 (21.05%)	48 (21.81%)	12 (18.46%)	0.681
Female	225 (78.94%)	172 (78.18%)	53 (81.53%)	
Mean age (years)	51.54 ± 16.39	53.07 ± 13.9	46.36 ± 14.73	0.0008
Histology				
Papillary classic	178 (62.45%)	134 (60.9%)	44 (67.69%)	0.025
Follicular-variant	86 (30.17%)	71 (32.27%)	15 (23.07%)	
Hürthle cell-variant	9 (3.15%)	9 (4.09%)	0	
Tall cell-variant	12 (4.21%)	6 (2.72%)	6 (9.23%)	
Tumor				
Mean size (mm)	16.39 ± 11.19	16.13 ± 11.82	17.27 ± 8.77	0.47
Unique	205 (71.92%)	160 (72.72%)	45 (69.23%)	0.001
Multifocal	80 (28.07%)	60 (27.27%)	20 (30.76%)	
Microcarcinoma	57 (20%)	47 (21.36%)	10 (15.38%)	0.2686
Locoregional infiltration	73 (25.61%)	46 (20.9%)	27 (41.53%)	0.001

**Table 2:** Total thyroidectomy: complications

	**Total**	**Group A**	**Group B**	***P***
Temporary hypoparathyroidism	80 (28.07%)	55 (25%)	25 (38.46%)	0.049
Permanent hypoparathyroidism	17 (5.96%)	10 (4.54%)	7 (10.76%)	0.117
Temporary unilateral vocal cord palsy	5 (1.75%)	3 (1.36%)	2 (3.07%)	0.698
Permanent unilateral vocal cord palsy	0	0	0	-
Bilateral vocal cord palsy	0	0	0	-
Neck hematoma	3 (1.05%)	2 (0.9%)	1 (1.53%)	0.798

### Pathological data

Median tumor size was 16 mm (16 mm in group A and 17 mm in group B). A microcarcinoma (<1 cm) was diagnosed in 57 patients (20%): 47 in group A (21.36%) and 10 in group B (15.38%). The histotype was classic papillary in 178 patients (62.45%, 134 in group A and 44 in group B), follicular-variant in 86 (30.17%, 71 in group A and 15 in group B), Hürthle cell-variant in 9 (3.15%, all in group A) and tall cell-variant in 12 (4.21%, 6 in group A and 6 in group B). Eighty patients (28.07%) had multifocal tumors: 60 in group A (27.27%) and 20 in group B (30.76%). Seventy-three patients (25.61%) had a locoregional infiltration (T3): 46 in group A (20.9%) and 27 in group B (41.53%) (see Table 1).

The two groups were well matched for sex, histology, tumor size, and radioiodine treatment; while the median age was slightly higher in group A, multifocality and locoregional infiltration were most represented in group B and incidence of microcarcinomas was most represented in group A. In group B, metastases were found in 17 patients (26.15%) and nodal micrometastases in 3 cases (4.61%). The median number of removed lymph nodes was 7.5. In patients with metastases, the median number of metastatic nodes was 3.5.

### Radioiodine ablation

After surgery, 259 patients (90.87%) underwent adjuvant radioiodine ablation (1,850 to 3,700 MBq-^131^I). Apart from lymph node involvement, indications for postoperative ^131^I treatment were: a tumor > 1 cm, extra-capsular thyroid invasion or locoregional extension, unfavorable histological subtype (follicular, diffuse sclerosing, or tall cell-variant papillary cancer), multifocal disease, or BRAF-positive tumor specimens. Twenty-six patients (9.12%) with tumor < 1 cm and without risk factor (lymph node involvement, extra-capsular invasion, unfavorable histological subtype, multifocal disease, or BRAF-positive) were not submitted to adjuvant radioiodine ablation. To obtain adequate levels of endogenous TSH (>30 mU/ml), that are associated with an increased radioiodine uptake, patients stopped L-T4 replacement three to four weeks before radioiodine treatment; when L-T4 withdrawal was not indicated, TSH stimulation was achieved using rhTSH (Thyrogen®, Genzyme, USA), according to standard protocols. Post-therapy whole-body scanning was performed four to seven days after radioiodine treatment.

Radioiodine ablation was performed in 194 patients in group A and all 65 patients in group B. In five patients of group B, two or more radioiodine administrations were needed; in all the other patients only one radioiodine administration was performed. Differences between the two groups were not statistically significant (*P* = 0.476). Median dose administered was 2,420 MBq-^131^I in group A and 2,600 MBq-^131^I in group B; so prophylactic neck dissection did not statistically correlate with radioiodine dose.

Median postoperative stimulated thyroglobulin was 1.0 ng/ml in group A and 1.2 in group B. This value was not used to determine radioiodine dose given the slight difference between the two groups.

### Recurrence

No patient developed distant recurrence during follow-up. After TT and radioiodine ablation, ipsilateral nodal (III to IV) recurrence was observed in 6 cases (2.1%): 4 in group A (1.81%) and 2 in group B (3.07%) (*P* = 0.89). Only one central (VI) node recurrence was observed in group A (0.45%). The demographic characteristics of patients with recurrence were the following: 2 male (47 and 34 years) and 4 females (median age 57.5 years; range 34 to 69). The mean elapsed time between intervention and lymph node recurrence was 15 months in group A (range 6 to 24) and 7.5 months in group B (6 and 9). Two patients had classic-variant, 1 had follicular-variant, 1 Hürthle cell-variant and 2 patients had tall cell-variant papillary carcinomas. In particular, this last variant was strongly associated with recurrence (*P* = 0.001; Table 3). Locoregional infiltration and multifocality were also associated to risk of recurrence (*P* = 0.005 and 0.009, respectively; Table 3). In all cases, a lateral and central lymph node dissection was performed, followed by another session of radioiodine ablation. Median number of removed nodes was 4; median number of metastatic nodes was 2.5. No complications were observed after reoperation.

**Table 3:** Risk factors of recurrence: gender, age and histology

	**Recurrence**	***P***
Gender		
Male	2/60 (3.33%)	0.81
Female	4/225 (1.77%)	
Mean age	49.83 ± 15.58 (versus 51.58 ± 14.35 non-recurrent)	0.768
Histology		*P* = 0.001
Papillary classic	2/178 (1.12%)	
Follicular-variant	1/86 (1.16%)	
Hürthle cell-variant	1/9 (11.11%)	
Tall cell-variant	2/12 (16.66%)	
Tumor		
Mean size	22.5 ± 6.62 (versus 16.26 ± 11.24 non-recurrent)	0.177
Unique	1/205 (0.48%)	0.009
Multifocal	5/80 (6.25%)	
Locoregional infiltration		0.005
Present	5/73 (6.84%)	
Absent	1/212 (0.47%)	

## Discussion

Lymph node metastases in level VI nodes in PTC are common, with macroscopically positive gross nodal disease being present in 10 to 30% of patients [10,30,31], while the incidence of clinically non-palpable disease is reported in 40 to 70% of patients [10,19,32]. However, the management and impact on the prognosis of this form of lymph node metastasis is unclear [10]. Nodal involvement in PTC has been shown in a number of retrospective studies to be associated with an increased risk of locoregional recurrence but not with overall survival [2,10,22,33,34]. Some studies support the concept that microscopically positive lymph nodes do not appear to progress to recurrence whether or not they are removed [24,25]. Other reports indicate that nodal dissection in differentiated thyroid cancer can advantageously decrease locoregional recurrence and improve survival [2,10,22,34]. Arguments in favor of prophylactic central node dissection are: the high incidence of lymph node metastases; the insufficient diagnostic accuracy of ultrasonography and intraoperative exploration in 1/3 of PTC; and the failure of ^131^I ablation in about 30% of cases, especially when enlarged lymph nodes greater than 1 cm are present [10,23,28].

High resolution cervical US is the most sensitive method for detecting metastatic lymph nodes as small as 2 to 3 mm. Unfortunately, although US has a high sensitivity and specificity in detecting lateral lymph node metastasis, it is less useful in the central compartment when the thyroid gland is still present [7,24,35,36]. Preoperative ultrasonography have high specificity and positive predictive value, but low sensitivity (40 to 70%) and negative predictive value (of only about 61%) for the detection of lymph node metastases in the central neck compartment [2,6,26,28]. Intraoperative clinical assessment of lymph nodes is also inaccurate and can be confounded by the presence of enlarged nodes related to Hashimoto’s thyroiditis [24].

Central node dissection increases the number of patients with undetectable Tg levels [10]. For example, one study found that the number of patients with basal Tg serum levels < 0.2 ng/ml during L-thyroxine suppression therapy was significantly higher among those who had undergone prophylactic central neck dissection [2]. Indeed, the addition of central lymph node dissection to total thyroidectomy is repeatedly reported to decrease serum levels of Tg and increase the rates of undetectable Tg [1,14,16]. Our experience does not confirm these data.

Another argument in favor of prophylactic central neck dissection is the lower associated rates of morbidity compared to those associated with reoperation [10,37]. Reoperative central lymph node dissection is more challenging and puts the recurrent laryngeal nerve and parathyroid glands at increased risk [17,26] due to increased scar tissue, edema and friability of the tissues together with distortion of the landmarks [38]; reoperative surgery has also been associated with a higher risk of postoperative hematoma [39].

A prophylactic central lymphadenectomy permits a better staging of central neck compartment lymph nodes, but further benefits remain to be demonstrated [10]. Authors report a 30% increase in the number of patients with T1 PTC (preoperatively considered to be N0), for whom ^131^I ablation was indicated following routine central and lateral nodal dissection demonstrating unexpected nodal metastases. The rational for this approach, in patients with tumors < 1 cm, is that positive lymph nodes are an indication for radioiodine ablation [10]. Lymphadenectomy seems to play a role in staging and may therefore be indicated when radioactive treatment would not normally be administered. When radioiodine treatment is advisable, routine lymph node dissection does not modify the treatment protocol, and is, therefore, not indicated [10]. Recent studies have suggested that approximately one-third of patients who have prophylactic central neck dissection may be upstaged [2,37], and as a consequence radioiodine therapy is used significantly more frequently in these patients [2]. Thus, patients undergoing central neck dissection also have a higher chance of receiving treatment for subclinical micrometastatic disease [2]. Indeed, prophylactic central neck dissection was found to result in an increased use of radioactive ^131^I and, ultimately, in more favorable outcomes [2]. Our results do not confirm these data.

Many endocrine surgeons doubt that routine lymph nodal dissection offers any real benefits to patients, arguing that the procedure is associated with higher morbidity, especially injury to the parathyroid glands (most frequently the lower) [10,17,22], with rates of transient hypoparathyroidism of 14 to 60%, permanent hypoparathyroidism of 3 to 11%, transient vocal cord paralysis of 3 to 7%, and permanent recurrent laryngeal nerve injury of 0 to 4% [16,19,27,29,40,41]. TT is associated with a low morbidity rate, and thanks to radioiodine treatment and TSH suppression therapy, the incidence of locoregional lymph node recurrence is low. The need for reoperation in the central compartment is uncommon, but it is generally accepted that reoperations in the cervical compartments are associated with greater risks of hypoparathyroidism and recurrent nerve palsy [1,24,42-44]. However, Kim *et al*. [45] reported a 0% incidence of new recurrent laryngeal nerve palsy and a 0.4% incidence of permanent hypoparathyroidism in a series of 20 patients who underwent reoperation of the central lymph node dissection for recurrent or persistent thyroid cancer; data which have since been confirmed by other authors [1,18] and in our experience. Reoperation (lymph node dissection) is not usually associated with higher morbidity, especially in cases of unilateral dissection, although hypoparathyroidism and recurrent laryngeal nerve injury have been observed in up to 14% and 9% of patients, respectively [10].

The low rate (2.1%) of lymph node recurrence following TT observed in the present study is in line with data reported by others [10]. In the present study, analysis of the recurrence patterns revealed that clinical recurrence most commonly occurred at level III and IV in both groups, regardless of central neck dissection. Interestingly, only one central (VI) node recurrences was observed, confirming that the absolute benefit of central neck dissection may be small. Our results are in agreement with other findings in the recent literature [46]. We did not observe any complications after reoperations, likely because of the small number of patients requiring a second surgical intervention.

The complication rates observed following prophylactic central neck dissection (group B) are, in our experience, high: a transient or definitive hypoparathyroidism was observed in 55 (25%) and 10 patients (4.54%), respectively, in group A; while 25 (38.46%) and 7 patients (10.76%) were diagnosed in group B, respectively (*P* = 0.049 and 0.117). Temporary recurrent laryngeal nerve paralysis was observed in 3 patients in group A (1.36%) and in 2 patients in group B (3.07%) (*P* = 0.69). In particular, the differences in the rate of transient hypoparathyroidism were statistically significant while the incidence of definitive hypoparathyroidism and temporary recurrent laryngeal nerve paralysis are approximately double in group B, even if the differences do not reach statistical significance, probably because of the limited number of cases.

To decrease the risk of postoperative complications related to prophylactic central neck dissection, unilateral central neck dissection has emerged as an alternative approach to bilateral central neck dissection [20,25]. Ipsilateral central neck dissection appears to be a safe, efficacious, and interesting alternative to bilateral central neck dissection, especially for small (≤1 cm) PTC, but no conclusive data are available [2,10,22,25,29]. Nevertheless, a bilateral central neck dissection was performed in all 65 cases of the present study, in view of the risk of skip and contralateral metastases [47].

As a matter of fact, the recent American Thyroid Association Guidelines, as well as a meta-analysis conducted by Chisholm *et al*. [9], whilst taking into consideration all the pros and cons, recommend routine central lymph node dissection for all differentiated thyroid cancers, especially in high-risk patients [10,22]. Prophylactic central neck dissection is not recommended for low-volume thyroid surgeons: the risk of nerve injury and hypocalcaemia appears to be significantly greater for low-volume centers [2,41].

Of consequence, the current indications for performing a prophylactic central neck dissection are still a matter of debate. Our low recurrence rate (2.1%) combined with the non-negligible incidence of complications (particularly transient and definitive hypoparathyroidism, 38.46% and 10.76% respectively) leads us to sustain that prophylactic central lymph node dissection should not be carried out on a routine basis in the treatment of PTC. Instead, it may be more useful to develop criteria for the identification of high risk patients for whom central neck dissection could be of benefit. The incidence of recurrence depends on numerous factors, such as tumor size, patient age, sex (males are more disposed to recurrence), and extra-capsular spread [2,9,22,42]. In our study, tumor histology was strongly associated with risk of recurrence: Hürthle cell-variant (11.11%) and, in particular, tall cell-variant (16.66%) carcinomas were associated with a high risk of relapse (*P* = 0.001). Multifocality (6.25%, *P* = 0.009) and extrathyroidal invasion (6.84%, *P* = 0.005) also presented a higher risk of recurrence, in line with other reports in the literature [2,9,22]. We found no differences in relation to age or gender, while smaller tumors were at lower risk of recurrence, in agreement with other reports in the literature [2,9,22]. Moreover, 20% patients presented microcarcinomas (tumors ≤ 1 cm in diameter) with low risk of recurrence, a rate that is again in agreement with the literature [48].

The problem that remains is how to define the assessment criteria of high-risk patients, considering the fact that only the size of the tumor can be assessed preoperatively, while the type and the histological characteristics (that is locoregional infiltration and multifocality) can usually only be identified after surgery. We believe that a prophylactic central neck dissection should not be routinely recommended for smaller tumors (≤1 cm) while it may be advisable for larger tumors (>2 cm), especially if cytological suspicion of a high risk subtype arises or if there are intraoperative signs of extra-capsular spread. A wider use of immunocytochemical and genetic markers could prove useful in better defining the high-risk population. For example, patients with RET/PTC oncogene expression have a higher rate of lymph node metastases [49], and this could constitute a useful factor to consider in the future. The development of techniques for the intraoperative identification of metastatic lymph nodes could also help the surgeon in this difficult choice.

## Conclusions

The role of prophylactic central lymph node dissection in the management of PTC remains controversial. There is no convincing evidence that prophylactic central neck dissection leads to an improvement in recurrence rate, overall survival, or any clinically significant variable when applied indiscriminately to all PTC patients. The recurrence rate found in this study of 2.1% confirms the rarity of lymph node recurrence and leaves many doubts regarding the usefulness of prophylactic central neck dissection. The lateral neck was mainly involved in recurrence regardless of initial central neck dissection, suggesting the clinical benefit of central neck dissection may be small.

Our study confirms that prophylactic central neck dissection is associated with increased morbidity, even when performed by experienced surgeons: in particular, it is associated with a higher rate of transient complications, mostly hypoparathyroidism.

Therefore, in our opinion, until conclusive evidence emerges of the actual benefit of prophylactic central neck dissection procedure in the treatment of PTC without suspicious enlarged nodes, it may be avoided.

In conclusion, TT appears to be an adequate treatment for clinically node-negative PTC. Prophylactic central neck dissection could be considered for the more appropriate selection of patients for radioiodine treatment and should be reserved for high-risk patients only. Unfortunately, no clinical or pathological factors are able to predict with any certainty the presence of nodal metastasis. However, in our experience, tumor size is related to an increased risk of recurrence, as are some histological types (Hürthle cell and, particularly, tall cell-variant), multifocality, and locoregional infiltration.

The potential use of molecular markers will hopefully offer a further strategy to stratify the risk of recurrence in patients with PTC and allow a more tailored approach to offer prophylactic central neck dissection to patients with the greatest benefit. Multi-institutional larger studies with longer follow-up periods are necessary to draw definitive conclusions.

## Abbreviations

FNAC: fine-needle aspiration cytology; PTC: papillary thyroid carcinoma; PTH: parathyroid hormone; rhTSH: recombinant human thyrotropin; TgAb: anti-Tg antibody; Tg: thyroglobulin; TSH: thyrotropin; TT: total thyroidectomy; US: ultrasound.

## Competing interests

The authors declare that they have no competing interests.

## Authors’ contributions

PGC conceived of the study and drafted the manuscript. GP participated in the design of the study and operated some of the patients. FM participated in the design of the study and performed the statistical analysis. JM helped to review the literature and collected patient follow-up data. LG collected patient follow-up data and helped to review the literature. EE participated in the design of the study and helped to draft the manuscript. AN participated in the design and coordination of the study and helped to draft the manuscript. All authors read and approved the final manuscript.
